# *Mycoplasma bovis* infections in Swiss dairy cattle: a clinical investigation

**DOI:** 10.1186/s13028-015-0099-x

**Published:** 2015-02-22

**Authors:** Marlis Aebi, Bart HP van den Borne, Andreas Raemy, Adrian Steiner, Paola Pilo, Michèle Bodmer

**Affiliations:** Clinic for Ruminants, Vetsuisse Faculty, University of Berne, Bremgartenstrasse 109a, PO Box 8466, , CH-3001 Berne, Switzerland; Veterinary Public Health Institute, Vetsuisse Faculty, University of Berne, Schwarzenburgstrasse 155, CH-3097 Liebefeld, Switzerland; Institute for Veterinary Bacteriology, Vetsuisse Faculty, University of Berne, Längassstrasse 122, PO box8466, , CH-3001 Bern, Switzerland

**Keywords:** Mycoplasma bovis, Mastitis, Pneumonia, Herd-level risk factors, Dairy herd

## Abstract

*Mycoplasma bovis* causes mastitis in dairy cows and is associated with pneumonia and polyarthritis in cattle. The present investigation included a retrospective case–control study to identify potential herd-level risk factors for *M. bovis* associated disease, and a prospective cohort study to evaluate the course of clinical disease in *M. bovis* infected dairy cattle herds in Switzerland. Eighteen herds with confirmed *M. bovis* cases were visited twice within an average interval of 75 d. One control herd with no history of clinical mycoplasmosis, matched for herd size, was randomly selected within a 10 km range for each case herd. Animal health data, production data, information on milking and feeding-management, housing and presence of potential stress- factors were collected. Composite quarter milk samples were aseptically collected from all lactating cows and 5% of all animals within each herd were sampled by nasal swabs. Organ samples of culled diseased cows were collected when logistically possible. All samples were analyzed by real-time polymerase chain reaction (PCR). In case herds, incidence risk of pneumonia, arthritis and clinical mastitis prior to the first visit and incidence rates of clinical mastitis and clinical pneumonia between the two visits was estimated. Logistic regression was used to identify potential herd-level risk factors for *M. bovis* infection. In case herds, incidence risk of *M. bovis* mastitis prior to the first visit ranged from 2 to 15%, whereas 2 to 35% of the cows suffered from clinical pneumonia within the 12 months prior to the first herd visit. The incidence rates of mycoplasmal mastitis and clinical pneumonia between the two herd visits were low in case herds (0–0.1 per animal year at risk and 0.1-0.6 per animal year at risk, respectively). In the retrospective-case-control study high mean milk production, appropriate stimulation until milk-let-down, fore-stripping, animal movements (cattle shows and trade), presence of stress-factors, and use of a specific brand of milking equipment, were identified as potential herd-level risk factors. The prospective cohort study revealed a decreased incidence of clinical disease within three months and prolonged colonization of the nasal cavity by *M. bovis* in young stock.

## Background

Bovine mycoplasmosis due to *Mycoplasma bovis* has caused major economic losses in the USA and Canada for several years, inducing mastitis [1,2], respiratory disease [3], and polyarthritis [4]. Both dairy and beef cattle have been affected [5]. *Mycoplasma* species most frequently associated with outbreaks of mycoplasmal mastitis in North America have been identified as *M. bovis,* followed by *M. alkalescens, M. bovigenitalium, M. californicum* and *M. canadense* [2]. To date, no effective therapy of mastitis and only limited success in treatment of respiratory and joint diseases [6,7] caused by *M. bovis* have been reported. Moreover, vaccines have not proved to be protective [3,8]. In Europe, reports have also documented the association between *M. bovis* and mastitis, pneumonia and polyarthritis [9-11]. Tschopp *et al.* [12] investigated respiratory infections in veal calves and reported that 50.3% of clinical respiratory episodes were attributable to *M. bovis.* In Switzerland, a seroprevalence study was performed by Burnens *et al.* [13] following an outbreak of *M. bovis*-associated pneumonia. That study showed a between-herd sero-prevalence of 47% and a cow level sero-prevalence of 6.1% in *M. bovis* antibody positive herds, implying that a large percentage of herds had been in contact with *M. bovis,* but only very few animals within the herds seroconverted. This pattern is different from other countries and production systems, such as feedlots where an overall *M. bovis* seroprevalence of 33.3% in one feedlot was observed [14]. In feedlots investigated in France, one *M. bovis* strain became predominant during the fattening period and was responsible for severe outbreaks of bovine respiratory disease (BRD) with high within-group prevalences [15]. Several authors [16,17] have suggested that mycoplasmas are frequently present in the cattle population, causing disease only if specific conditions are met. Such circumstances can occur if animals’ immune response is impaired due to stress such as inadequate feeding, transportation or low environmental temperatures [17,18]. Punyapornwithaya *et al.* [19] found that the colonization of different body sites with one specific *M. bovis* strain was responsible for a mastitis epidemic within a herd. Additionally, hematogenous spread of specific strains may occur after infection of one organ system [20]. Most recently, Spergser *et al.* [21] described an outbreak of *M. bovis* on a communal Alpine farm caused by one specific strain. During the last 2 to 3 years, incidental field reports have indicated an increase in number of outbreaks due to *M. bovis* in Swiss dairy cattle herds, mainly associated with the development of mastitis and pneumonia.

The aim of this study was to collect clinical and epidemiological data on mycoplasmal outbreaks in Swiss dairy cattle herds and to identify potential herd-level risk factors associated with clinical *M. bovis*-related disease using a retrospective case–control study. Additionally, the course of mycoplasmal disease in *M. bovis* infected herds that received recommendations for improvement was assessed using a prospective cohort study design.

## Methods

### Herd selection

In order to recruit herds with mycoplasmosis, the project had been announced to routine diagnostic laboratories by letter and practicing veterinarians in Switzerland by e-mail and announcements at seminars for continuous education. From April 2010 till October 2011, affected herds were reported. These herds, which had at least one animal with clinical signs of mastitis, pneumonia or arthritis and at least one *M. bovis* polymerase chain reaction (PCR) or culture positive sample in materials such as milk, broncho-alveolar lavage fluids, nasal swabs, lung or mammary gland-tissue during the last 3 months before study enrollment were included in the study and were defined as case herds.

To have balanced study groups, one control herd was selected for each case herd taking into account the herd size and geographical distance to the case herd. The Swiss Animal Movement Database was consulted to identify five neighboring herds located within a 5 km range from the case herd and with a similar herd size (+/−10 cows). If no herds were located within 5 km of the case herd, the radius was increased to 10 km. From this list, one herd was randomly selected and contacted with a request to participate. These herds were defined as control herds, given the additional requirement that they did not have a history of clinical disease caused by *M. bovis* within the past 12 months, which was reported by the farmer and confirmed by the attending veterinarian, respectively.

### Definition of clinical cases

Clinical pneumonia in cows and calves was defined as a condition with elevated body temperature, elevated respiratory rate, pathological lung sounds and coughing. A case of clinical mastitis was defined by abnormal changes in milk secretion and/or swelling of affected quarters with or without systemic symptoms such as elevated body temperature. Subclinical mastitis was defined as a somatic cell count >150,000 cells/ml without changes in secretion or swelling of affected quarters. Arthritis was defined as distinct swelling of one or more joints with or without lameness.

### Collection of herd data and clinical samples

During the first and second visits to case and control herds, the same structured questionnaire was used to assess farm data. At each herd visit, housing, milking system, milking routine and calf management were evaluated. The questions concerned general farm information covering the topics housing system, animal movements (trade, cattle shows) and milking system. The age-specific number of cases with a clinical diagnosis of pneumonia or arthritis within the last 12 months was recorded with the aid of treatment records for young stock or adults separately. Mastitis cases caused by *M. bovis*, as confirmed by either PCR or bacteriological culture within the 3-months-period prior to the first visit, were also recorded. Milking hygiene and milking routine were observed during milking and included sequence of work steps during udder preparation, presence of a milking order according to udder health status of the individual cows, and post milking teat disinfection. Intensive animal movements (animal traders and herds with >5 transportations per year) and presence of concomitant disease within the previous 12 months such as puerperal metritis, displacement of abomasum, post-partum hypocalcaemia (“milk fever”) and clinical ketosis were assessed by consulting farm records. Overcrowding, high in-barn temperature (>24°C) and impaired feed quality were listed as stress-factors. Overcrowding was regarded as present if not all animals of one housing-unit (i.e. one row of cows in a tie stall, one deep straw box for group housing of calves) were able to lie down at the same time including a cubicle-to-cow ratio <1 for herds kept in loose housing systems. Feed quality was assessed by visual and olfactory inspection for presence of molds and for secondary fermentation of silages, respectively. An overview of the data collected on farms and management is given in Table 1.

**Table 1:** **On-farm questionnaire: overview on the data collected on the farms**

**Topic**	**Description**
Cow husbandry	Housing system (free-stall vs. tie-stall), lying area (rubber mats with saw dust, rubber mats with chopped straw, straw bedding, chalk-straw bedding, other), flooring (rubber mats, concrete, slatted floor, other),
Calf husbandry	Group housing vs. individual igloos, bedding type (deep straw bedding vs, other bedding), separate from lactating cows (yes vs. no)
Heifer rearing	Own replacements (yes vs. no), rearing on another farm (yes vs. no)
Communal alpine pasturing	Heifers only (yes vs. no), cows and heifers (yes vs. no)
Feeding management, ration of lactating cows	Changes in the ration within the last 3 months (yes vs. no), changes in feeding management (yes vs. no)
Quality of feed fed to cows	Smell (aromatic vs. moldy, alcoholic), heating of silage (by hand), visual inspection (presence of molds), changes in feed quality within the last 3 months (yes vs. no)
Feeding management of calves	Bucket feeding (individual bucket vs. one bucket for several calves), automatic feeding system (yes vs. no), feeding mastitis milk to calves (yes vs. no)
Milking system	Type of milking system (parlor vs. bucket milk unit vs. pipeline system), brand of milking system, changes in the milking system within the last 3 months (yes vs. no)
Milking routine	Grouping of mastitic and susceptible cows, milking routine (order and implementation of individual steps), teat cleaning material (no cleaning, disinfectant towel, dry paper towel, wood wool, other), post milking teat disinfection, (yes vs no), changes in milking routine within the last 3 months (yes vs. no)
Animal movement	Number of purchased animals within the last 12 months, sporadic transportations (<5 transportations per year), expositions (>5 movements per year), animal trade
Overcrowding	All animals in one housing unit can lie down at the same time (yes vs. no), cubicle to cow ratio 1:1 or higher in loose housing systems (yes vs. no)
Environmental factors	Temperature > 24°C (yes vs. no), building work in progress (yes vs. no)
Concomitant disease in animals suffering from confirmed mycoplasmosis	Retained fetal membranes, hypocalcaemia, puerperal metritis, displacement of abomasum, primary ketosis, lameness caused by claw disorders

At the first and second herd visit, composite foremilk samples were aseptically collected from all lactating cows of case and control herds during milking time. Dry nasal swabs (Transwab Amies, Medical Wire & Equipment, Wiltshire, UK), were collected from all age groups in both case and control herds as a convenience sample from at least 5% of cows present at the visit, including healthy animals, in order to detect subclinical carriers of *M. bovis*. The nasal swabs were transported to the laboratory without any transport medium. Additionally, lung and mammary gland tissues of clinically diseased cows that were culled were collected from the abattoir if logistically possible. All samples were transported at 5°C to the Institute of Veterinary Bacteriology, Vetsuisse Faculty Berne for *M. bovis* analysis.

For the prospective cohort study, farmers from case herds were given general advice on how to proceed with *M. bovis* suspected and positive animals and how to optimize their management concerning mastitis and pneumonia to avoid new infections of *M. bovis* susceptible animals, based on literature [2,21-23]. The recommendations included isolating and culling all positive animals with clinical mastitis. Also, isolating and treating animals with symptoms of severe pneumonia and positive nasal swabs with oxytetracyclin (Oxysentin, Novartis, Basle, Switzerland, 10 mg/kg per day, intravenously) for at least 7 days was advised, in addition to applying non-steroidal anti-inflammatory drugs (NSAID). The advice to improve milking hygiene and milking routine included the implementation of a strict milking order: 1. low somatic cell count cows, with no clinical signs of pneumonia or arthritis, 2. suspect cows either with unclear *M. bovis* status or signs of pneumonia and polyarthritis, and 3. *M. bovis* positive cows (milk). Udder preparation should include the following steps in the given order: 1. fore stripping; 2. cleaning of teats (1 towel/cow); 3. attachment of clusters after a preparation-lag time of 60–90 s; 4. removal of clusters with interruption of vacuum, and 5. post-milking teat disinfection with a disinfecting solution, preferably containing iodophores, immediately after cluster removal. To prevent new *M. bovis* infections in young animals, the owners were advised to house all young stock separately from the cows, to isolate calves with pneumonia, to avoid feeding calves with milk from cows with mastitis and to use individual feeding buckets. On-farm pasteurization of raw milk as described by Butler *et al.* [24] to feed dairy calves is not routinely practiced in Switzerland and was therefore not considered as a potential control measure in this study.

Eighteen case herds were visited a second time within a mean interval of 75 d (SD 20 d) from the first visit in order to retest all lactating cows for the presence of *M. bovis*, to assess ongoing transmission within the herd and to test first calving heifers and cows that had been dried off at the first visit. One case herd was not visited a second time since it was owned by an animal trader and most of the cows were sold or moved to other farms belonging to the same owner at the time of the second visit.

The cohort study focused on the course of clinical *M. bovis*-associated disease in case herds. Nevertheless, 9 control herds were visited a second time and investigated using the same structured questionnaire in order to confirm freedom from *M. bovis*. Sampling in case and control herds was performed according to the same protocol used at the first visit, except for nasal swabs where a different convenience sample of animals was used. Farmers were encouraged to send in milk samples for PCR analysis of cows suspected of mycoplasmal mastitis between the two herd visits and to take the rectal temperature and call their veterinarians for cows suspected of clinical pneumonia. No further investigations on pathogens were recommended.

### Laboratory testing

In order to identify *M. bovis* positive animals, milk, nasal swabs and organs were tested for *M. bovis* by a direct real-time PCR as previously described by Rossetti *et al.* [25]. Briefly, lysates from samples were prepared as DNA template [25,26]. Fifty μl of milk sediment was added to 200 μl of lysis buffer (0.1 M Tris–HCl, pH 8.5, 0.05% Tween 20, 0.24 mg/ml proteinase K) and swabs taken from gross lesions in organs and nasal swabs were dipped for 1 min in 500 μl of lysis buffer. All lysates were incubated for 1 h at 60°C and 15 min at 95°C as a denaturation step. To identify *M. bovis* in samples, a real-time PCR specific for *M. bovis* and targeting the *uvrC* gene was further performed using a Ct cutoff value of 40 [25,26]. This method detects approximately 2,000 colony-forming units in the sediment of 1 ml of milk and does not cross react with *M. agalactiae, M. capricolum* subsp. *capricolum, M. capricolum* subsp*. capripneumoniae*, *M. conjunctivae*, *M. hyopneumoniae*, *M. leachii*, *M. putrefaciens, M. mycoides* subsp*. capri*, *M. mycoides* subsp. *mycoides* or 19 other bacterial species [25].

### Statistical analysis

Raw data were checked for completeness and correctness and corrected if needed. Observations with missing values were removed from further analyses. Statistical analyses were performed using SAS 9.3 (SAS Institute, Cary, USA). In order to gather information on the long-term history of included case and control herds, incidence risks of pneumonia and arthritis were retrospectively estimated for the 12-months-period prior to the first herd visit. The incidence risk of *M. bovis*-associated mastitis was estimated including cases confirmed by PCR or bacterial culture up to 3 months prior to the first visit only. The incidence rate of clinical *M. bovis*-associated mastitis between the two herd visits was estimated for the lactating cow population at risk. Since all herds had an all year round calving pattern, the number of lactating cows was estimated by subtracting 14% from the annual mean number of cows to exclude the dry cow population according to Kretzschmar *et al.* [27]. Incidence rates of clinical pneumonia were estimated separately for adult cows and young stock. In order to describe the course of disease potentially related to *M. bovis* after the first visit, incidence rates were estimated by dividing the number of clinical cases by the animal years at risk, which was estimated by multiplying the mean number of animals by the length of the interval between the two visits. Incidence rates were expressed as the number of clinical *M. bovis* cases per animal year at risk.

To verify whether matching criteria of case and control herds concerning the herd size were met, the two groups were compared by cross tabulations. In order to assess whether case herds with a high prevalence of *M. bovis* mastitis also had a high prevalence of pneumonia in cows and calves, the Wilcoxon rank test was used.

Epidemiological data collected during the first visits in case and control herds, respectively was analyzed using univariable logisitic regression in order to identify potential herd-level risk factors associated with a *M. bovis* infection based on the Type 3 test for the case–control study design. Correlation between potential herd-level risk factors was assessed using a Spearman rank correlation matrix. Correlation was assumed to occur when the absolute Spearman rank correlation coefficients were >0.50. Significance level was set at *P* < 0.1 because of low statistical power.

The study was conducted in accordance with the animal welfare legislation of Switzerland, all (including ethical) aspects of the study had been previously approved by the Federal Food Safety and Veterinary Office. All farmers were thoroughly informed about the project prior to the herd visit and gave their informed consent for the sampling of milk specimens from their animals and for completion of a questionnaire regarding herd management practices.

## Results

### Analysis of samples

A total of 1,293 milk samples and 200 nasal swabs were collected at the first herd visit in case and control herds and analyzed by real-time-PCR. An overview of analyzed samples in case herds is given in Table 2. A total of 18 (2.4%; 95%-CI: 1.5-3.8%) milk samples collected in case herds during the first visit were *M. bovis* PCR positive and were collected from cows suffering from clinical mastitis with the typical symptoms of severe swelling and changes of secretion followed by spreading of the clinical signs to all four quarters. All milk samples collected during the first herd visit from control herds were PCR negative (Table 3). A total of 138 nasal swabs were collected during the first visit from case herds of which 44 (31.9%, 95%-CI: 24.7-40.1) were PCR positive for *M. bovis*. Except for one transiently PCR-positive nasal swab of one clinically healthy calf, there were no *M. bovis* positive animals found in control herds (Table 3). The calf was re-examined and retested by a nasal swab within 50 days, showing no clinical signs and a negative PCR result. The collection of nasal swabs of 5% of cows present was not always reached, because adequate restraint of adult cows was not possible in all housing systems and required more assistance than available. Additionally, young stock (other than calves younger than 3 months) was not present on all farms visited during the summer months because of seasonal alpine pasturing.

**Table 2:** **Overview of collected samples in 19 case herds with at least one clinical case caused by** ***Mycoplasma bovis*** **within a three-month-period prior to the first herd visit**

	**Samples tested visit 1**				**Samples tested visit 2**				**Additional samples (positive)**		
	**Milk**		**Nasal swabs**		**Milk**		**Nasal swabs**				
**Case herd ID**	**No**	**PCR pos.**	**No**	**PCR pos.**	**No**	**PCR pos.**	**No**	**PCR pos.**	**Milk (visit 1-visit 2)**	**Lung**	**Mammary gland**
1	28	3	3	1	25	0	0	0	0	1	0
2	46	1 (4)*	8	0	37	0	0	0	3	0	1
3	8	1	3	1	9	0	3	0	0	0	0
4	46	1	10	0	42	0	5	0	0	0	0
5	44	1	10	8	45	0	8	5	1	2	1
6	32	0	4	1	36	0	2	0	0	1	0
7	19	1	2	1	19	0	5	1	1	0	1
8	63	1	0	0	62	0	8	7	0	0	0
9	34	0	16	4	40	0	11	5	0	0	0
10	60	4	9	4	57	0	4	0	10	0	0
11	33	0	12	2	38	0	8	0	0	0	0
12	50	3	13	7	40	0	7	1	0	0	1
13	87	0	6	3	0	0	0	0	0	0	0
14	49	2	3	3	38	0	3	0	0	0	0
15	19	0	16	6	20	0	8	1	0	0	0
16	15	0	7	1	17	0	5	4	0	0	0
17	52	0	5	1	49	0	8	1	0	1	0
18	20	0	7	1	16	0	8	1	0	0	0
19	37	0 (1)*	4	0	40	0	0	0	0	0	0
**Total**	**742**	**18**	**138**	**44**	**630**	**0**	**93**	**26**	**19**	**5**	**4**

*Number of samples analyzed shortly before the first herd visit in two different external diagnostic laboratories (Suisselab AG, Zollikofen, Switzerland, Department for Clinical Microbiology and Infection Biology, Faculty of Veterinary Medicine, Vienna, Austria).

**Table 3:** **Overview of collected samples in 17 control herds during the first and second herd visit**

	**Samples tested visit 1**				**Samples tested visit 2**			
	**Milk**		**Nasal swabs**		**Milk**		**Nasal swabs**	
**Control herd ID**	**No**	**PCR pos.**	**No**	**PCR pos.**	**No**	**PCR pos.**	**No**	**PCR pos.**
1	21	0	1	0	21	0	0	0
2	40	0	3	0	42	0	4	0
3	7	0	2	1	7	0	1	0
4*	36	0	0	0	0	0	0	0
5*	30	0	8	0	0	0	0	0
6*	18	0	6	0	0	0	0	0
7	18	0	2	0	19	0	2	0
8*	60	0	0	0	0	0	0	0
9*	32	0	6	0	0	0	0	0
10	58	0	4	0	54	0	4	0
11	46	0	9	0	38	0	0	0
12*	49	0	0	0	0	0	0	0
13	57	0	5	0	50	0	2	0
14	16	0	4	0	18	0	4	0
15	10	0	3	0	9	0	2	0
16*	25	0	4	0	0	0	0	0
17*	28	0	5	0	0	0	0	0
**total**	**551**	**0**	**62**	**1**	**258**	**0**	**19**	**0**

*Herds were not visited a second time.

During the second visit to the case and control herds, none of the cows showed the typical clinical signs of *M. bovis* mastitis, and none of the milk samples tested positive for *M. bovis* (Tables 2 and 3). In contrast, 28.0% (26/93) PCR-positive nasal swabs were collected in 60% (9/15) of case herds, at the second herd visit, which was not significantly different from the prevalence at the first herd visit (*P* = 0.52).

Clinical signs of pneumonia in five calves were only present in one case herd and no clinical pneumonia cases were found in adult cows during the second visit.

### Description of herds and herd-level risk factors

A total of 23 case herds and 17 control herds were visited during the study period. Four case herds were excluded from the case–control study because of insufficient data, leaving 19 case and 17 control herds, available for statistical analyses. No adequate control herds could be found for two case herds. For the purpose of the prospective cohort study, 18 case herds and 9 control herds were visited a second time. No control herds had to be excluded because of a recent *M. bovis* history. The mean herd size of case and control herds was 41.1 (SD = 19.1) and 34.4 (SD = 16.1) lactating cows, respectively (*P* = 0.26). Mean milk production in case herds was 8,316 kg (SD = 1050.7) and 7,605.9 kg (SD = 1089.9) in control herds and differed significantly (*P* = 0.07). The following breeds were present in the study herds: Brown Swiss (4 herds), Simmental (2 herds), Swiss-Fleckvieh-Red Holstein (19 herds), and Holstein (7 herds). Three herds kept a mixture of breeds.

An overview of the risk factors is given in Table 4. Assessment of milking routine revealed that case herds applied 4.7 and 7.5 times more often fore-stripping and additional stimulation until milk let down, respectively, than control herds. On case premises, cows were 15.0 times more often milked with a specific brand of milking equipment and they also had a higher milk production than cows in control herds. Stress-factors possibly affecting the immune system including high in-barn temperature, frequent animal movement (trade or >5 transportations per year), moldy feed, overcrowded barn and concomitant disease were 5.6 and 4.4 times more often identified in cows and calves of case herds respectively compared to cows and calves of control herds. Finally, case herds had 8.3 times more animal movements due to trade and cattle shows than control herds. The variables fore-stripping and additional stimulation until milk ejection were correlated (Spearman rank correlation coefficient was 0.51); other risk factor pairs were not.

**Table 4:** **Risk factors associated with the herd-level presence of** ***Mycoplasma bovis*** **in univariable logistic regression models**

**Variable**	**Category**	**Frequency**	**Frequency and prevalence (%) of case herds**	**OR**	**90% confidence interval**		***P*** **- value**
					**lower**	**upper**	
Brand of milking equipment*	Special brand Other brands	14 21	12 (85.7) 6 (28.6)	15.0	3.4	66.3	0.003
Milk Production of herd (increase of 100 kg)	Continuous	Mean = 79.7, SD = 11.1		1.067	1.006	1.13	0.07
Presence of stress factors cows**	Yes No	16 20	12 (75.0) 7 (35.0)	5.6	1.6	18.9	0.02
Presence of stress-factors calves***	Yes No	9 27	7 (77.8) 12 (44.4)	4.4	1.01	18.9	0.1
Animal movement (trade and exposition)	Yes No	12 24	10 (83.3) 9 (37.5)	8.3	2.0	35.5	0.02
Fore-stripping	Yes No	25 11	16 (64.0) 3 (27.3)	4.7	1.3	17.5	0.05
Additional stimulation until milk ejection	Yes No	30 6	18 (60.0) 1 (16.7)	7.5	1.1	50.3	0.08

Significance was set at *P* < 0.1.

*Information on brand of milking system is missing for one farm.

**Stress-factors cows include: overcrowding, moldy feed, high in-barn temperature, frequent transportation, concomitant disease.

***Stress-factors calves include: overcrowding, concomitant disease, high degree of calf traffic.

Implementation of the following recommendations to decrease *M. bovis*-associated disease was recorded during the second visit to case farms. Eighteen of nineteen case farmers culled the clinically diseased animals within approximately one week from the herd visit and two case farmers changed their milking routine. One farmer switched to a new cleaning material and the other used a different dipping solution. None of the case farmers isolated the diseased animals and no changes were made in calf-housing.

### The course of clinical disease associated with M. bovis

**Figure 1:**
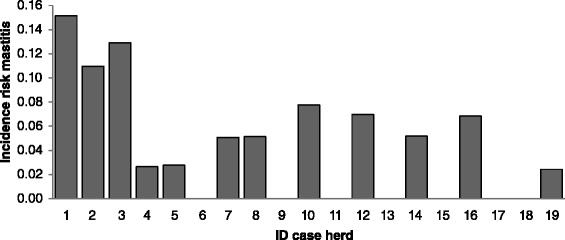
**Incidence risk of** ***Mycoplasma Bovis*** **-associated mastitis in case herds in the three month-period prior to the first herd visit.**

**Figure 2:**
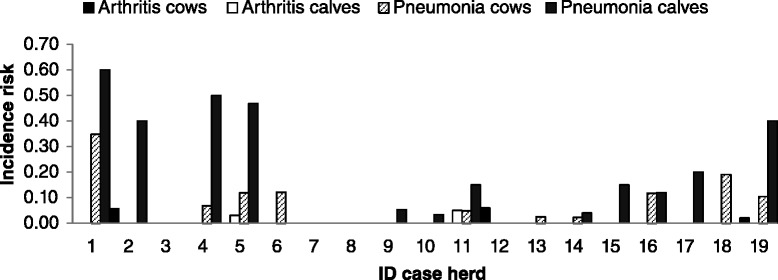
**Incidence risk of pneumonia and arthritis in calves and cows (according to the definitions given in the material and methods section) in case herds in the 12-month-period prior to the first herd visit.**

**Figure 3:**
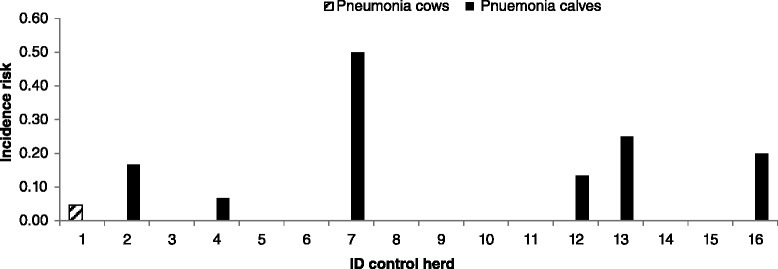
**Incidence risk of pneumonia in calves and cows (according to the definition given in the material and methods section) in control herds 12-months prior to the first visit.** Data on the exact number of calves of control herd 10 was not available; therefore incidence risk of pneumonia in calves could not be estimated for this herd.

In 12 case herds (63%), clinical mastitis was the predominant clinical symptom, whereas in the remaining seven case herds (37%), the predominant clinical finding was pneumonia in cows. In case herds, the incidence risk of *M. bovis* mastitis within 3 months prior to the first visit ranged from 0.01 to 0.13 (Figure 1). Arthritis was observed in three case herds, otitis media/interna in calves in one case herd. Within-herd incidence risk of pneumonia in cows, pneumonia in calves, arthritis in cows and arthritis during the 12-month-period prior to the first visit are shown in Figure 2 and ranged from 0 to 0.60 in case herds. The incidence risk of clinical pneumonia in calves and cows in control herds is displayed in Figure 3.

Case herds reported significantly more cases of clinical pneumonia during the 12-month-period prior to the first herd visit in cows (*P* = 0.006) and calves (*P* = 0.1), respectively, than control herds. Additionally, no differences in the herd rankings for incidence risk of *M. bovis*-associated mastitis and the herd rankings for incidence risk of clinical pneumonia in calves (*P* = 0.64) or in cows (*P* = 0.97) could be identified.

Incidence rate estimation of clinical mastitis and clinical pneumonia in the prospective cohort study (i.e., between the two visits) revealed that only in three case herds new clinical cases occurred: Case herd number 1 had a pneumonia incidence rate in cows of 0.6 per animal year at risk but no new mastitis cases were identified. Case herd number 11 had an incidence rate of clinical pneumonia in cows of 0.3 per animal year at risk. Case herd number 5 had a clinical pneumonia incidence rate in cows of 0.1 per animal year at risk and a clinical mastitis incidence rate of 0.1 per animal year at risk. In case herds 1 and 11, incidence rates of clinical pneumonia in calves were 0.2 and 0.1 per animal year at risk, respectively.

Incidence rate of clinical pneumonia in cows and calves and incidence rate of mastitis due to *M. bovis* in the nine control herds visited a second time was 0 per animal year at risk. Information on disease of young stock that was seasonally pastured on alpine farms was not available.

## Discussion

### Retrospective case–control study: herd-level risk factors

The rather low prevalence of animals with clinical signs of *M. bovis*-associated disease supports the hypothesis that *M. bovis* is an opportunistic pathogen that needs certain circumstances to induce clinical disease as previously suggested [17]. The herd-level risk factors identified in this study may contribute to these circumstances. A high rate of animal movements because of trade or showing was associated with clinical disease due to *M. bovis* for instance. Contact of susceptible animals with infected animals outside the herd of origin and stress due to transportation and cattle shows may explain this. Interestingly, replacement purchase was not associated with being a case herd in our study and suggested elsewhere [2,16,17,22]. This might be due to the low rate of purchased animals in the respective herds and the low statistical power of the study. On the other hand, typing of *M. bovis* strains from all case herds was done in another study [26] and showed the presence of herd-specific strains. This implies that contact to other infected animals as the source of infection is unlikely. Moreover, it has been suggested that the stress of transport itself rather than contact to other animals (exposure to “new” strains) constitutes the risk factor [28]. Also, a high rate of animal movements particularly the visit to cattle shows might be related to the production level of the herds.

Besides frequent transportation, other stress-factors such as moldy feed, overcrowding in the barn and concomitant disease were present significantly more often in case herds than in control herds. Fink-Gremmels in 2007 [29] reviewed the impact of various mycotoxins on health and performance of dairy cows and concluded that interaction of mycotoxins can impede their rumen degradation and be a threat to metabolic, hormonal and immunological function of exposed individuals. It was also shown that the fusarium toxin deoxynivalenol present in naturally contaminated feed impaired nonspecific immunity by decreasing phagocytic function of neutrophils [30]. Additionally, case herds had a higher average milk production than control herds as previously described by Feenstra *et al*. [31]. This may indicate that cows from case herds were at a higher risk to be in a negative energy balance if not fed adequately and, therefore, might have been more susceptible to infectious diseases [32,33].

Case herds practiced significantly more often fore-stripping and additional stimulation until proper milk let down, indicating a more appropriate milking routine than applied in control herds. There seems to be no obvious biological reason why fore-stripping should increase the risk of *M. bovis* associated mastitis and has to be interpreted with care due to the limited statistical power in the present study. However, manual stimulation of the udder and the teats before attachment of the milking clusters is accompanied by a more intensive contact of the potentially contaminated milkers’ hands with the teat ends and may increase the risk of infection of the mammary gland. Reverse causality of the factor “fore-stripping” could be excluded in all case herds since changes in milking routine after the start of the outbreak were only introduced in two herds. It included the usage of a different cleaning material in one herd and the introduction of post milking teat disinfection in the other herd. It is therefore not assumed that farmers of case herds applied fore-stripping to control the *M. bovis* outbreak.

The strongest risk factor identified in the univariable logistic regression models was “brand of milking equipment”. This factor has to be interpreted as a confounder for being a *M. bovis* herd, because the estimates of other explanatory variables changed >20% when this variable was added to multivariable logistic regression models [34] (data not shown).

### Descriptive results and prospective cohort study

In 17 out of 19 case herds, a high incidence risk of clinical pneumonia in cows and calves was present within the one-year-period prior to the outbreak when compared to control herds. Case herds with a high incidence risk of clinical pneumonia in cows or calves in the 12- month-period prior to the first visit also suffered from the highest incidence risk of *M. bovis*-associated mastitis within the three-month-period prior to the first visit. Although the involvement of *M. bovis* in the pneumonia cases recorded during the 12-month-period prior to the first visit was not confirmed by bacteriological examination, it can be hypothesized that *M. bovis* was already present in the case herds for some time without causing additional disease such as mastitis and arthritis [16]. Personal experience from former herd investigations (MB) have shown that the incidence of clinical pneumonia in adult cattle is often increased prior to an outbreak of *M. bovis*-associated mastitis. The time period of three months for inclusion of *M. bovis* associated clinical mastitis cases was also based on experience from former investigations of infected herds (MB). Within case herds, the prevalence of *M. bovis*-associated mastitis varied between 0.01 and 0.13, which agrees with previous results [23].

Arthritis in adult cattle was only observed in three herds and with very low incidence risks (0.06, 0.06 and 0.02 respectively). *M. bovis*-associated arthritis in adult cattle is often described as sporadic [3]. In contrast to dairy herds, prevalence of the chronic pneumonia and polyarthritis syndrome of up to 28% were reported in Canadian feedlot cattle [5], which might be attributable to commingling of animals from different herds of origin increasing the risk of social stress and introduction of pathogens.

The possible spread of *M. bovis* by feeding contaminated milk to calves has been reported previously [23,24,35], and recently, Maunsell *et al.* [36] demonstrated that colonization of tonsils occurred after oral inoculation of calves with *M. bovis*. This might play an important role under Swiss conditions, where young calves and cows are often housed in the same barn, and replacement calves are fed almost exclusively with unpasteurized milk. *M. bovis* is also capable of forming biofilms on smooth surfaces (glass) and microtitre plates [37] and it might therefore also be able to survive on drinking nipples, which could be an important fomite for the spread of mycoplasmas when feeding several calves with the same drinking equipment. This, however, could not be statistically confirmed in the present study.

When interpreting incidence rates of disease attributable to *M. bovis* between the first and second visit, there were only three case herds reporting additional cases of mastitis (case herds 5 and 7) and pneumonia (case herds 1 and 5). Case herd 5 had a major problem with overcrowding in the barn, and cows and calves were housed close to each other. Additionally, the owner of case herd 5 neither isolated nor culled one of the cows with a confirmed clinical mastitis and pneumonia due to *M. bovis*, which might have contributed to the prolonged course of clinical disease in this herd. The prevalence of clinical mastitis, pneumonia and polyarthritis in cows due to *M. bovis* at the second visit decreased to 0% in all the case herds visited twice (n = 18). This indicates that clinical disease was either self-limiting or that implementation of management improvement was lowering the within-herd transmission of *M. bovis,* especially in the case of intramammary infection, as reported previously [2,21-23,38]. Randomized field trials are needed to determine the true effectiveness of these interventions.

### Limitations

Since the most important limitation of the present study was the small sample size, an additional multivariable logistic regression analysis correcting for confounding and co-linearity could not be completed because of insufficient statistical power. Therefore, the results of the univariable regression analysis have to be interpreted with caution. We are also aware that in control herds potentially infected animals might have been missed since an insufficient number of nasal swabs was collected. However, none of the nasal swabs and none of the milk samples of the nine revisited control herds were positive for *M. bovis.* Despite these limitations, except for the risk factor “fore-stripping” and the confounding factor “use of a specific brand of milking equipment”, the significant risk factors can be explained biologically.

## Conclusions

Using a combination of study designs, this investigation identified some interesting aspects of *M. bovis* infection in Swiss dairy cattle herds. In the retrospective case–control study, risk factors such as high milk production, presence of frequent animal movements, moldy feed and overcrowding were associated with an outbreak of clinical *M. bovis* disease. The prospective part of the investigation showed an improvement in the herds’ *M. bovis* status but a prolonged colonization in clinically healthy animals was also identified. Young stock, as a reservoir for *M. bovis*, should be taken into account in future control strategies.
